# Caveolin-1 Is Up-Regulated by GLI1 and Contributes to GLI1-Driven EMT in Hepatocellular Carcinoma

**DOI:** 10.1371/journal.pone.0084551

**Published:** 2014-01-13

**Authors:** Xiaohong Gai, Zhongtang Lu, Kangsheng Tu, Zheyong Liang, Xin Zheng

**Affiliations:** Department of Hepatobiliary Surgery, the First Affiliated Hospital of Xi'an Jiaotong University, Xi'an, Shaanxi, China; Philipps University, Germany

## Abstract

Caveolin-1 (Cav-1) has been recently identified to be over-expressed in hepatocellular carcinoma (HCC) and promote HCC cell motility and invasion ability via inducing epithelial-mesenchymal transition (EMT). However, the mechanism of aberrant overexpression of Cav-1 remains vague. Here, we observed that Cav-1 expression was positively associated with GLI1 expression in HCC tissues. Forced expression of GLI1 up-regulated Cav-1 in Huh7 cells, while knockdown of GLI1 decreased expression of Cav-1 in SNU449 cells. Additionally, silencing Cav-1 abolished GLI1-induced EMT of Huh7 cells. The correlation between GLI1 and Cav-1 was confirmed in tumor specimens from HCC patients and Cav-1 was found to be associated with poor prognosis after hepatic resection. The relationship between protein expression of GLI1 and Cav-1 was also established in HCC xenografts of nude mice. These results suggest that GLI1 may be attributed to Cav-1 up-regulation which plays an important role in GLI1-driven EMT phenotype in HCC.

## Introduction

Caveolin-1 (Cav-1) is the first identified marker of caveolae (a kind of 50- to 100-nm cell membrane invagination[1]) which is also known caveolin/VIP21[2]. Cav-1 has been found to exist widely in a variety of tissue cells including adipocyte, endothelia and muscle cells[3]. Caveolae is enriched in signal molecules such as Src tyrosine kinases[4], small GTPase[5] and G protein[6]. Generally, Cav-1 functions as scaffolding protein to concentrate various ligands within caveolae and interact with them and in turn the relevant pathways were inhibited. Therefore, Cav-1 plays an important role in signal transduction. There are a growing body of studies about Cav-1 expression in cancer, and interestingly, it was found to be aberrantly increased in some kinds of malignances such as bladder cancer[7], esophagus carcinoma[8], T cell leukemia[9], and prostate cancer[10], whereas down-regulated in breast cancer[11], cervix cancer[12], lung cancer[13], sarcoma[14], ovarian cancer[15], thyroid follicular cancer[16] and colon cancer[17].

Recent studies showed that Cav-1 expression was increased significantly in HCC tissues compared to normal liver tissues and liver cirrhosis tissues[18]–[21]. However, the role of Cav-1 on the progression of HCC remains controversial. Overexpression of Cav-1 was found related with metastasis and poor prognosis of HCC by several groups, which indicates Cav-1 acts as onco-protein in HCC pathogenesis[19]–[21]. On the other hand, there was a literature reporting that increased Cav-1 was correlated with prolonged overall survival of HCCs apparently[22], by which Cav-1 was considered as a HCC repressor. Although there are several studies paying attention to the effect of Cav-1 overexpression on HCC, limited investigation attempted to elucidate the underlying mechanism of Cav-1 overexpression in HCC.

Cokakli et al. verified that Cav-1 could promote migratory and invasive capacity of HCC cells through inducing epithelial-mesenchymal transition (EMT)[18]. EMT is a critical, highly conserved process which controls cell differentiation and embryo development. A line of evidences have revealed that EMT modulates malignant characteristics of cancer cells such as mobility, invasion, anti-apoptosis and stem-liking phenotypes[23]. Our previous studies showed that EMT appeared frequently in HCC and was involved in increased migration and invasion ability of HCC cells[24], [25]. In addition, we demonstrated that GLI1 overexpression was responsible for EMT phenotype of HCC and indispensable for TGFβ1-driven EMT of HCC cells[24]. GLI1 is an important member of GLI transcription factor family which controls transcription of various downstream genes of Hedgehog pathway. In our preliminary investigation, GLI1 was found aberrantly up-regulated in HCC and predicted worse outcome of HCCs after liver resection.

Here, we attempted to address the following question: 1. What is the relationship between Cav-1 expression and postoperative survival of HCCs? 2. Does GLI1 leaded to up-regulation of Cav-1 in HCC? 3. Is Cav-1 involved in the GLI1-driven EMT of HCC cells?

## Results

### Cav-1 Promoted HCC Cell Migration and Invasion through Inducing EMT

**Figure 1:**
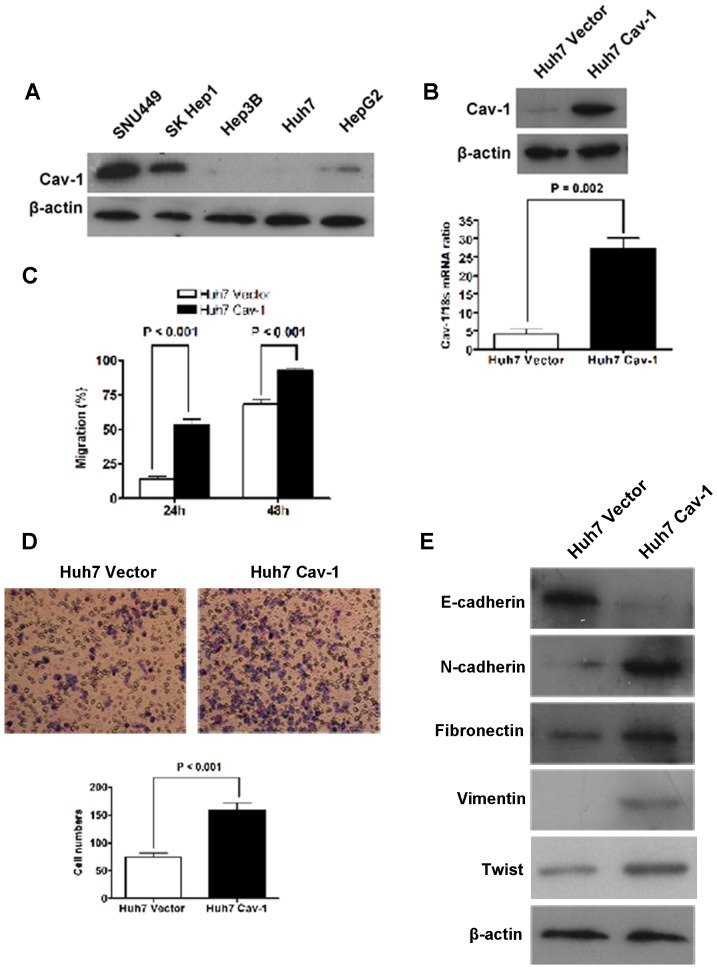
There were different level of Cav-1 expression in all five HCC cell lines and forced expression of Cav-1 leaded to up-regulation of cell migration and invasion and EMT phenotype of Huh7 cells. (A) The Cav-1 expression was different in five kinds of HCC cell lines; (B) Cav-1 expression in Huh7 cells was confirmed to be increased significantly after transfected with Cav-1 expressing plasmid by both qRT-PCR and Western immunoblotting; (C) As assessed by the wound healing assay, cell migration rate was increased in Huh7 cells by enforced expression of Cav-1 at both 24 and 48hours; (D) As assessed by Millicell invasion chamber assay, invasion capacity of Huh7 cells was increased apparently after overexpression of Cav-1; (E) After overexpression of Cav-1, E-cadherin expression was repressed and expression of mesenchymal markers including N-cadherin, Fibronectin and Vimentin was enhanced clearly.

Cav-1 expression was examined in five HCC cells. Western immunoblotting assay showed that both SNU449 cells and SK Hep1 cells expressed Cav-1 protein at high level, while there was limited expression of Cav-1 in HepG2 cells, Huh7 cells and Hep3B cells (Fig. 1A). Thus, we increased Cav-1 expression in Huh7 cells via transfecting Cav-1 expressing plasmid stably. Overexpression of Cav-1 was confirmed by both qRT-PCR and Western immunoblotting (Fig. 1B). As shown in Fig. 1C, the results of wound healing assay showed that the migration rate of Huh7 Cav-1 cells was significantly higher than that of Huh7 Vector cells at both 24 and 48hours. And Matrigel invasion assay showed that 158±13 Huh7 Cav-1 cells per field went across the Matrigel gel and chamber filter, while there were only 74±8 Huh7 Vector cells crossing the Matrigel gel (Fig. 1D). To figure out the relationship between Cav-1 and EMT, we assessed expression of EMT markers including E-cadherin, N-cadherin, Fibronectin and Vimentin by Western immunoblotting and found that forced expression of Cav-1 decreased E-cadherin expression and increased expression of N-cadherin, Fibronectin and Vimentin (Fig. 1E). In addition, Twist which has been considered as the EMT inducer was also found up-regulated in Huh7 after overexpression of Cav-1 (Fig. 1E), while other well-known EMT inducers including Slug and SNAI1 weren't found affected by overexpression (Figure S1A).

**Figure 2:**
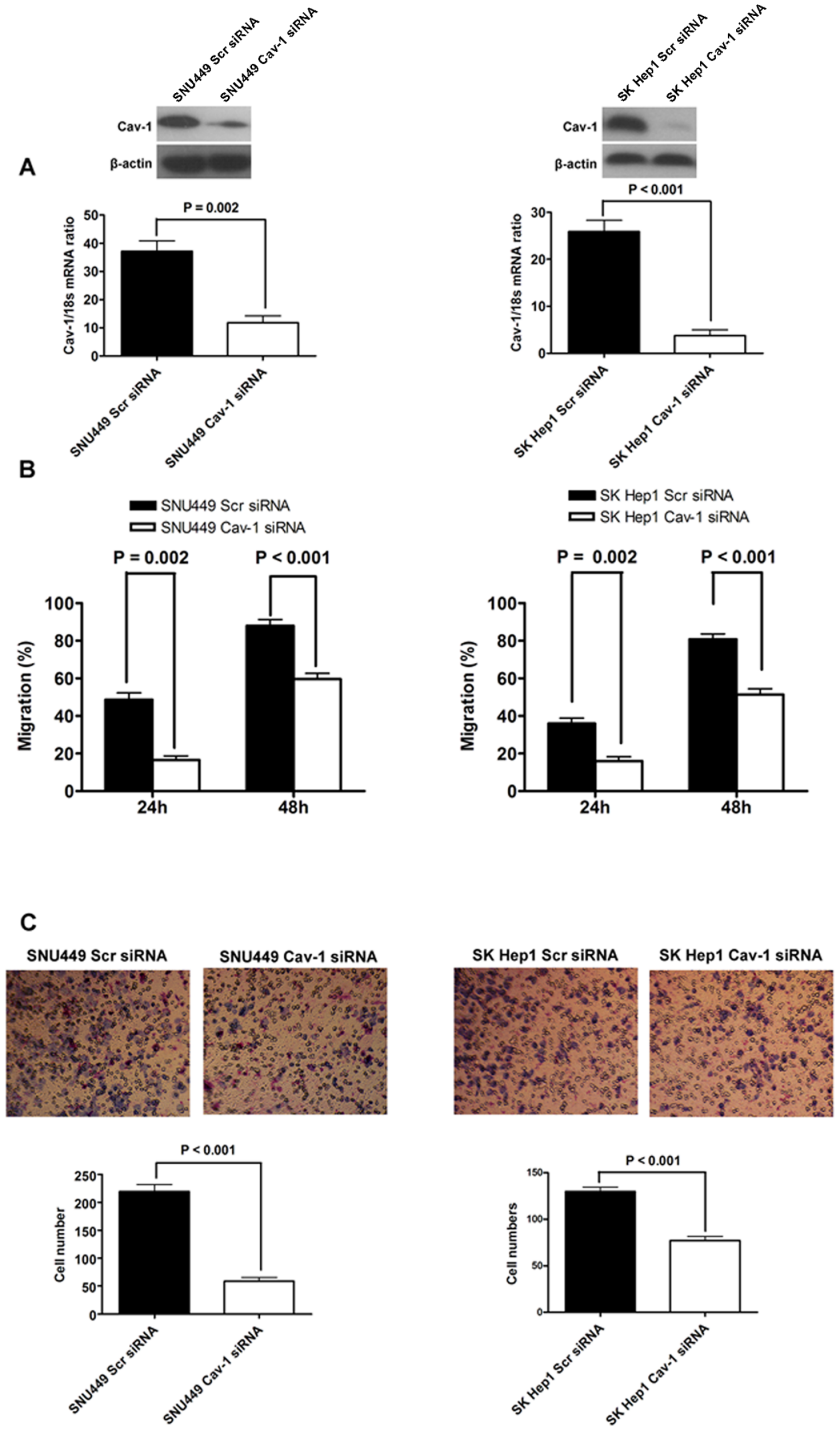
Silencing Cav-1 abated migration and invasion abilities of HCC cells. (A) Cav-1 siRNAs were confirmed to decrease expression of Cav-1 in both SNU449 and SK Hep1 cells by both qRT-PCR and Western immunoblotting; (B) As assessed by the wound healing, knockdown of Cav-1 inhibited cell migration of both SNU449 and SK Hep1 cells; (C) Silencing Cav-1 attenuated invasion ability of both SNU449 and SK Hep1 cells, as assessed by Millicell invasion chamber assay.

**Figure 3:**
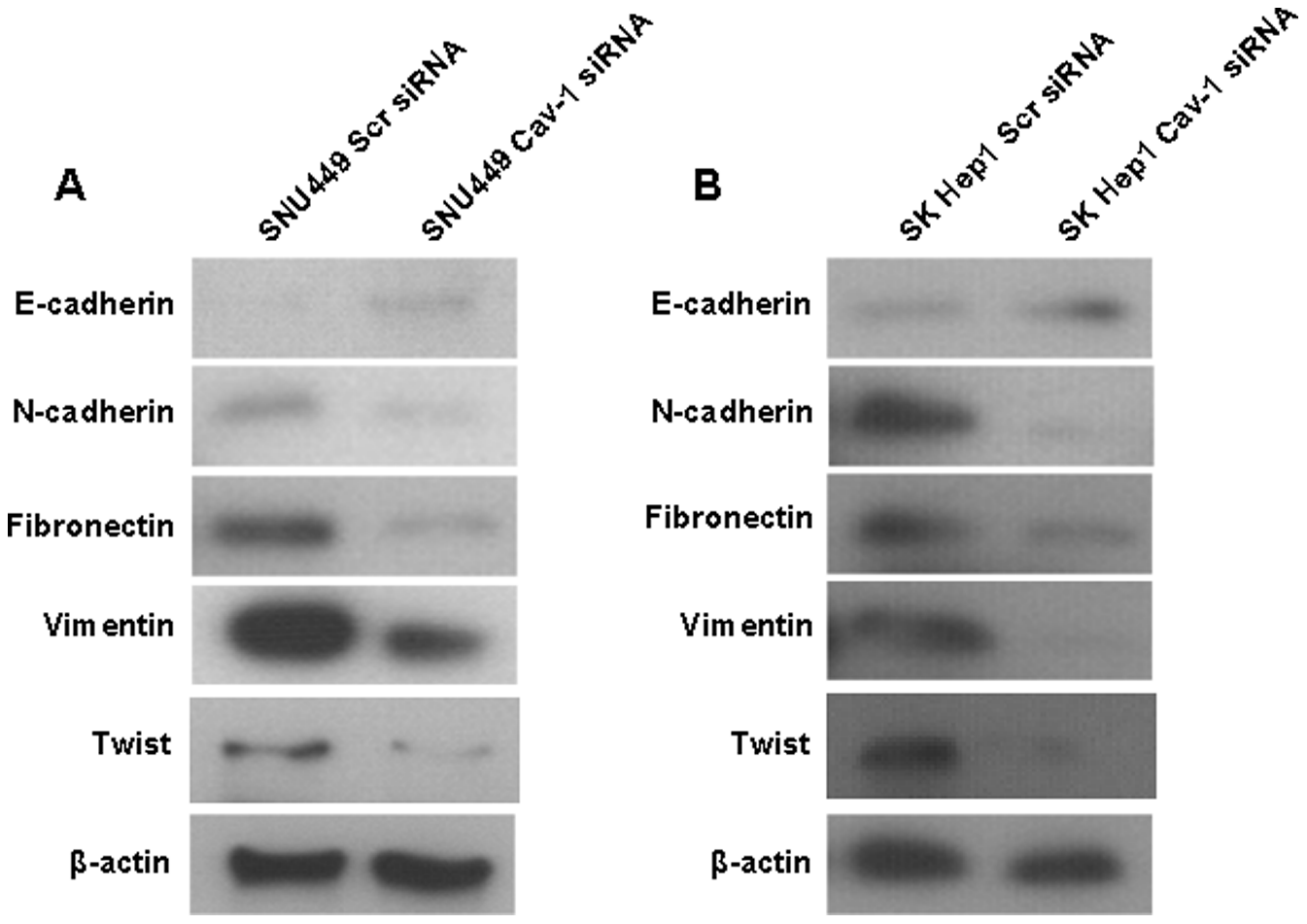
Knockdown of Cav-1 reversed EMT phenotype of HCC cells. (A) Knockdown of Cav-1 leaded to E-cadherin up-regulation and down-regulation of mesenchymal markers including N-cadherin, Fibronectin and Vimentin in SNU449 cells, as assessed by Western immunoblotting; (B) Silencing Cav-1 was found to increase E-cadherin expression and decreased the expression of N-cadherin, Fibronectin and Vimentin in SK Hep1 cells by Western immunoblotting.

To evaluate the effect of Cav-1 on migration, invasion and EMT phenotype of HCC cells, we silenced Cav-1 expression of SNU449 cells via siRNA sequences from Santa Cruz Biotechnology. The mRNA and protein expression of Cav-1 were verified to be decreased greatly after transfection with Cav-1 siRNAs (Fig. 2A). As shown in Fig. 2B, wound healing assay showed that SNU449 cells transfected with Cav-1 siRNAs (SNU449 Cav-1 siRNA) had significantly lower mobility than scrambled siRNA-transfected controls (SNU449 Scr siRNA). There were less cells passing through Matrigel gel and chamber filter in SNU449 Cav-1 siRNA group than that in SNU449 Scr siRNA group (P<0.001, Fig. 2C). As assessed by western immunoblotting, E-cadherin protein expression was enhanced while expression of N-cadherin, Fibronectin and Vimentin was decreased in SNU449 Cav-1 siRNA cells compared with SNU449 Scr siRNA cells (Fig. 3A). In consistent with the results of Cav-1 overexpression experiments, knockdown of Cav-1 repressed Twist expression (Fig. 3A and B) and didn't influence the expression of both Slug and SNAI1 in SNU449 cells (Figure S1A), which verified further that Twist took part in Cav-1-driven EMT of HCC cells.

These results indicate that Cav-1 enhances mobility and invasion of HCC cells via inducing EMT. To validate this finding, we also transfected Cav-1 siRNA into SK Hep1 cells and found that expression of Cav-1 was silenced successfully (Fig. 2A). Both migration and invasion of SK Hep1 cells transfected with Cav-1 siRNAs (SK Hep1 Cav-1 siRNA) were suppressed clearly (Fig. 2B and C). In addition, knockdown of Cav-1 was found to enhance E-cadherin expression and repress expression of mesenchymal markers including N-cadherin, Fibronectin and Vimentin by Western immunoblotting (Fig. 3B). We also evaluated the regulatory effect of Cav-1 on expression of EMT inducers including Twist, Slug and SNAI1 and found that knockdown of Cav-1 repressed Twist expression (Fig. 3) and didn't affect expression of both Slug and SNAI1 in SK Hep1 cells (Figure S1A).

To confirm the influence of Cav-1 ablation on cell migration and EMT phenotype of HCC cells, we used another pool of Cav-1 siRNAs including three different siRNA sequences from GenePharma Co. to repeat the mentioned above assessments. As shown in Fig. S2A, Cav-1 expression was silenced successfully in SNU449 cells by siRNAs. Similarly, Cav-1 knockdown also resulted in remarkable down-regulation of migration and invasion capacity of SNU449 cells (Figure S2B and 2C). These Cav-1 siRNA constructs also leaded to up-regulation of E-cadherin in SNU449 cells, while decreasing the expression of N-cadherin, Fibronectin and Vimentin (Figure S2D). Finally, Twist expression was found repressed in SNU449 cells by these Cav-1 siRNA sequences. Taken together, these data suggested strongly that Cav-1 induced EMT phenotype through up-regulating Twist expression and consequently accelerated cell migration and invasion in HCC cells.

### Cav-1 Was Up-regulated by GLI1 and Attributed to GLI1-driven EMT in HCC

**Figure 4:**
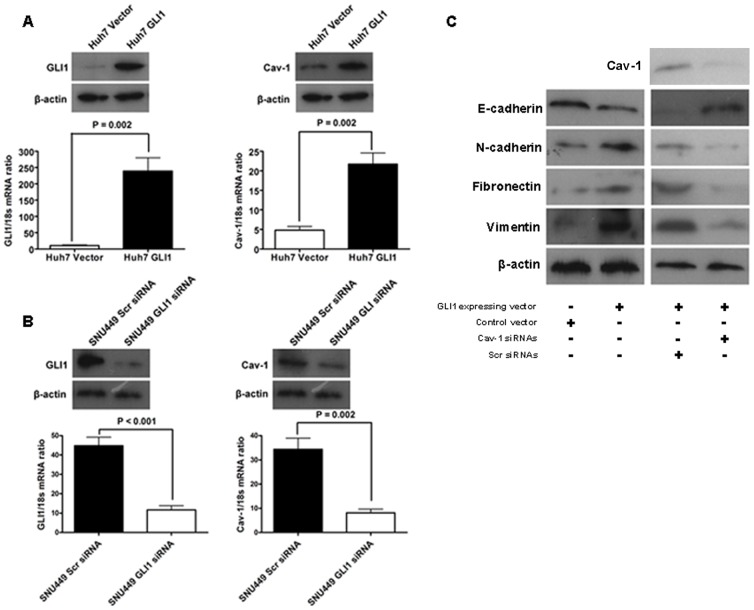
Cav-1 was up-regulated by GLI1 and contributed into GLI1-driven EMT phenotype of HCC cells. (A) As examined as both qRT-PCR and Western immunoblotting, enforced expression of GLI1 in Huh7 cells (left) increased Cav-1 expression (right); (B) Both qRT-PCR and Western immunoblotting revealed that knockdown of GLI1 in SNU449 cells (left) resulted in down-regulation of Cav-1 (right); (C) The results of Western immunoblotting showed that ectopic expression of GLI1 induced down-regulation of E-cadherin and up-regulation of N-cadherin, Fibronectin and Vimentin in Huh7 cells. When we repressed the Cav-1 up-regulation induced by GLI1 overexpression in Huh7 cells, E-cadherin expression was increased and the expression of N-cadherin, Fibronectin and Vimentin was attenuated apparently.

In our preliminary studies, we found that GLI1 was able to induce EMT and accelerated mobility and invasion of HCC cells. Accordingly, we proposed the hypothesis here that GLI1 might regulate Cav-1 expression and induce EMT in HCC cells. To test it, we enforced GLI1 expression in Huh7 cells which expressed both GLI1 and Cav-1 at low level using GLI1 expressing plasmid and found that forced expression of GLI1 up-regulated Cav-1 in Huh7 cells (Fig. 4A). In consistent with our previous data[24], GLI1 overexpression induced suppression of E-cadherin and up-regulation of N-cadherin, Fibronectin and Vimentin (Fig. 4C), which indicates that GLI1 leads to EMT of Huh7 cells. On the other hand, we abolished GLI1 expression of SNU449 cells with high levels of both GLI1 and Cav-1 using GLI1 siRNAs (Fig. 4B). Knockdown of GLI1 resulted in down-regulation of Cav-1 in SNU449 cells (Fig. 4B). The positive association between GLI1 and Cav-1 in HCC cell lines prompted us to search the Cav-1 promoter for the potential GLI1 binding sites. After searching −1,961 bp upstream of the Cav-1 transcriptional start site, no consensus GLI1 binding site (5′-GACCACCCA-3′) was found in this region. However, bioinformatics analysis demonstrated the presence of two candidates of GLI1 binding site. The first potential site located −115/−123 bp upstream of the GLI1 transcriptional start site and its homology to the canonical GLI1 binding site was about 66.7%. Another potential GLI1 binding site (−1340∼−1348 bp) also had 66.7% homology to the canonical one (Figure S3A). Then, we obtained Cav-1-pGL3 reporter vector which was synthetized by combining −1,961 bp upstream of the Cav-1 transcriptional start site with pGL3 basic luciferase reporter vector from Genomeditech Co. (Shanghai, China) and transfected this reporter vector into both Huh7 GLI1 cells and Huh7 Vector cells. With the help of the Dual-Luciferase Reporter assay from Promega, it was found that there was significantly more reporter activity in Huh7 GLI1 cells than one in Huh7 Vector cells (Figure S3B). However, on account of these two potential GLI1 binding sites, we didn't get positive results of chromatin immunoprecipitation (ChIP). These results suggested strongly that Cav-1 was regulated positively by GLI1 in HCC cells.

To determine whether Cav-1 plays an important role in GLI1-driven EMT of HCC cells, we inhibited Cav-1 up-regulation induced by forced expression of GLI1 in Huh7 cells through transfecting Cav-1 siRNAs (Fig. 4C) and found it leaded to up-regulation of E-cadherin and down-regulation of N-cadherin, Fibronectin and Vimentin in Huh7 GLI1 cells (Fig. 4C), which states clearly that inhibition of Cav-1 reverses EMT phenotype of HCC cells induced by GLI1.

In addition, to determine whether Cav-1 could mediate any other aspects of GLI function besides induction of EMT, we assessed whether Cav-1 could modulate the markers of Hh signaling activation including SNAI1 and PTCH1 which are also the GLI1 target genes by western immunoblotting. The results showed that neither overexpression of Cav-1 in Huh7 nor knockdown of Cav-1 in SNU449 cells and SK Hep1 cells affected expression of PTCH1 and SNAI1, as shown in Figure S1A. In additional, the Hh pathway reporter assay also showed that neither overexpression of Cav-1 nor knockdown of Cav-1 affected the activation of Hh pathway in HCC cells (Figure S1B). Hence, it seems that Cav-1 up-regulated by GLI1 couldn't activate Hh signaling further in HCC cells.

### Cav-1 Was Overexpressed in HCC Tissues and Predicted Poor Postoperative Outcome

**Figure 5:**
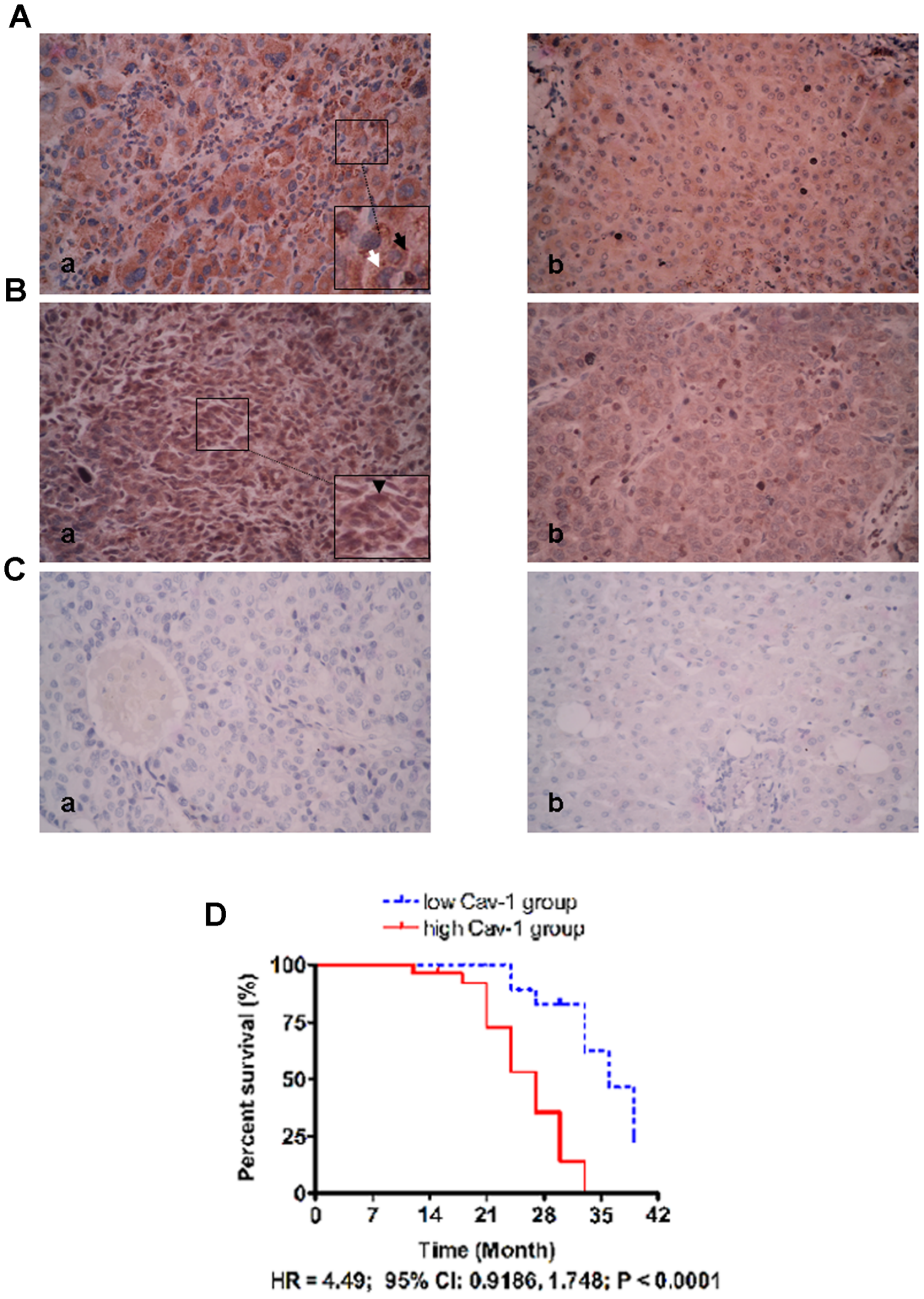
Overexpression of Cav-1 was frequently found in HCC tissues, which predicted poor prognosis of HCCs after liver resection. (A) The representative IHC staining of Cav-1 in HCC tissues (a) and adjacent normal tissues (b). There was Cav-1 protein expressing in both cell membrane (as labelled by black arrows) and cytoplasm (as labelled by a white arrow); (B) The representative IHC staining of GLI1 in HCC tissues (a) and adjacent normal tissues (b). Most of GLI1 protein located in cell nucleus, as shown by black arrow head; (C) The negative control staining in HCC tissues (a) and adjacent liver tissues (b); (D) HCC patients with higher level of Cav-1 had the worse prognosis after hepatic resection than ones with lower level of Cav-1 (HR = 4.49, P<0.0001).

To find out expression of Cav-1 in both HCC tissues and adjacent liver tissues, we carried out IHC staining with primary antibody against Cav-1. The results showed that the positive immunoreactivity was correlated with about 82% of HCC cases (65/80), while only 40% adjacent liver tissues (32/80) showed positive protein staining. The Cav-1 protein was found expressed in both cell membrane (as labelled by black arrows) and cytoplasm (as labelled by a white arrow) of HCC cells by IHC assay (Fig. 5A). After analysis of the results of IHC scores using Mann-Whitney test, we found that Cav-1 expression in HCC tissues was significantly higher than one in adjacent tissues (P<0.01). The representative results were shown in Fig. 5A. To evaluate the relationship of protein expression between Cav-1 and GLI1 in HCC tissues, we also performed IHC to detect GLI1 expression in HCC tissues and found that GLI1 was also up-regulated in HCC tissues and positive associated with Cav-1 significantly in HCC tissues (Fig. 5B, r  = 0.420, P<0.05) after analyzing the IHC scores using the Spearmen rank test, which suggests that GLI1 could lead to up-regulation of Cav-1 in HCC tissues. IHC assay showed that GLI1 protein mainly located in cell nucleus (Fig. 5B, as shown by black arrow head).

To examine whether the level of Cav-1 in tumor tissues helps to predict survival of HCCs after liver resection, we followed up the HCC patients recruited in this study. We obtained the follow-up information of 58 HCC cases (72.5%). The median duration of follow up was 27 months (range: 9–39 months). These 58 patients were classified into two groups by Cav-1 expression in HCC tissues, the low Cav-1 group and high Cav-1 group using the median IHC score of Cav-1 in HCC tissues as the cut-off value. After analyzing them with the Kaplan-Meier survival curve, we found that patients with high Cav-1 expression had apparently worse postoperative outcome than ones with low Cav-1 expression (as shown in Fig. 5D, HR  = 4.49; 95% Cl: 0.9186, 1.748; P<0.0001). These results suggest that Cav-1 expression is aberrantly increased in HCC tissues and predicts poor prognosis after liver resection and there is a positive relationship between Cav-1 and GLI1 protein expression in HCC tissues, which is consistent with the result of in *vitro* experiments of this study.

### There Was Positive Correlation between Protein Expression of Cav-1 and GLI1 in Huh7 Xenografts of Nude Mice

**Figure 6:**
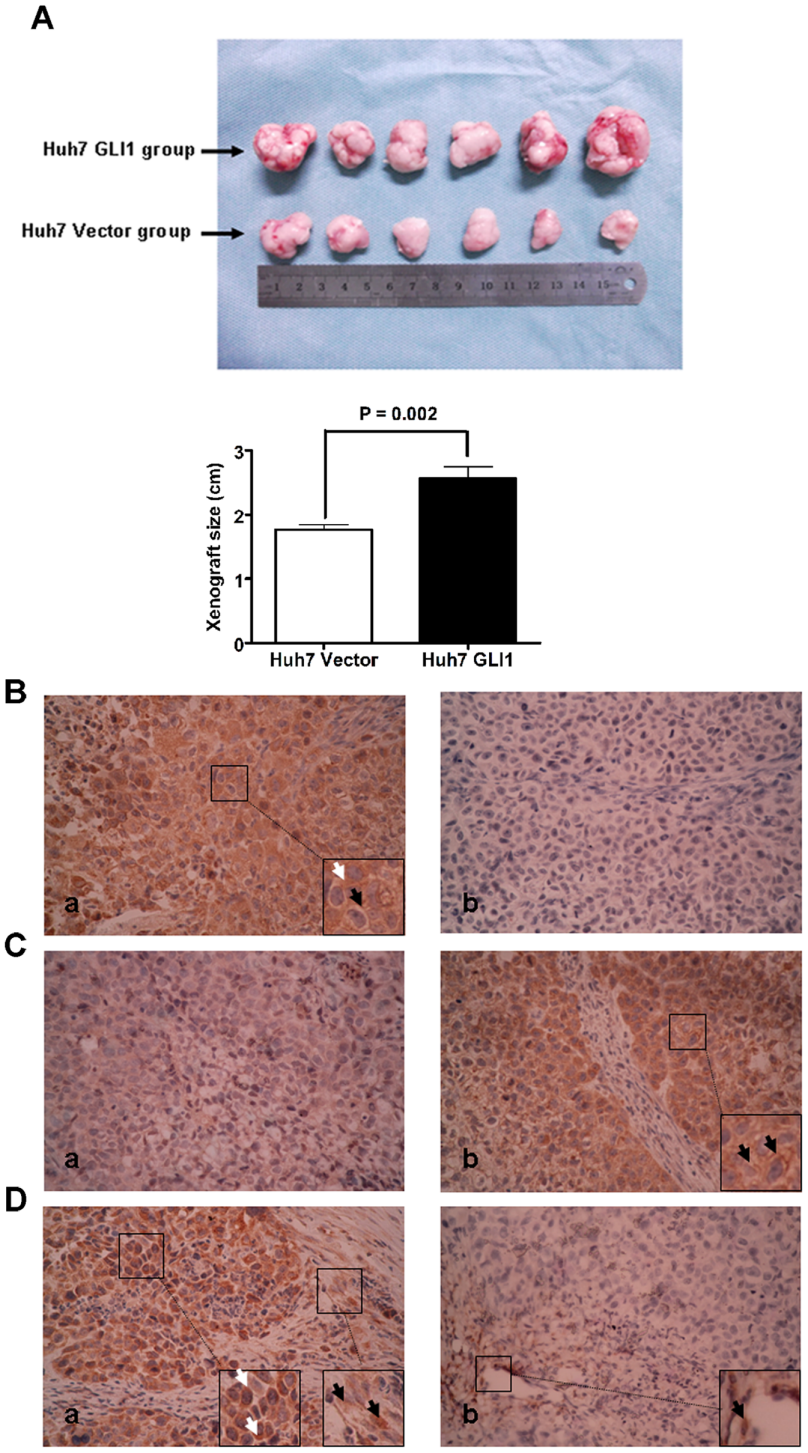
GLI1 was found to promote growth of Huh7 xenografts and result in more Cav-1 expression and EMT phenotype in Huh7 xenograft tissues. (A) The size of xenografts from Huh7 GLI1 group was significantly larger than one from Huh7 Vector group (P = 0.002); (B) The representative IHC staining of Cav-1 in xenografts from both Huh7 GLI1 group (a) and Huh7 Vector group (b). Cav-1 protein was detected in both cell membrane (as labelled by a black arrow) and cytoplasm (as labelled by a white arrow). Cav-1 expression in xenografts from Huh7 GLI1 group was apparently more than one in xenografts from Huh7 Vector group; (C) The representative IHC staining of E-cadherin in xenografts from both Huh7 GLI1 group (a) and Huh7 Vector group (b). E-cadherin protein located mainly in cell membrane, as labelled by black arrows. There were less E-cadherin expression in xenograft tissues from Huh7 GLI1 group than ones from Huh7 Vector group; (D) The representative IHC staining of N-cadherin in xenografts from both Huh7 GLI1 group (a) and Huh7 Vector group (b). N-cadherin expression expressing basically in cytoplasm (labelled by white arrows) was increased clearly in xenograft parenchymal tissues from Huh7 GLI1 group than ones from Huh7 Vector group. Cytoplasmic N-cadherin expression was also found in xenograft mesenchymal tissues from both groups, as labelled by black arrows.

To further prove our *in vitro* findings that GLI1 up-regulated Cav-1 in HCC, we performed the HCC xenograft mouse experiment. As shown in Fig. 6A, the size of xenografts derived from Huh7 GLI1 cells (Huh7 GLI1 group) was significantly larger that one from Huh7 Vector cells (Huh7 Vector group) at the 15th day after HCC cells injection. Additionally, we carried out IHC assay on xenograft tissues and found that Cav-1 was expressed positively in all six xenografts from Huh7 GLI1 group and there was negative expression of Cav-1 found in xenografts from Huh7 Vector group (as shown in Fig. 6B). The Cav-1 protein in xenografts from Huh7 GLI1 group was detected in both cell membrane (as labelled by a black arrow) and cytoplasm (as labelled by a white arrow), which is consistent with the finding of IHC assessment for HCC patients. E-cadherin expression was dramatically enhanced in xenografts Huh7 Vector group and mainly in cell membrane (Fig. 6C). As shown in Fig. 6D, N-cadherin was found strongly positive in xenografts from Huh7 Cav-1 group and expressed mainly in cytoplasm (as labelled by white arrows). We also found positive staining of N-cadherin in tumor mesenchymal tissues from both Huh7 Cav-1 group and Huh7 Vector group (as labelled by black arrows), whereas no positive staining of N-cadherin was detected in tumor parenchymal tissues from Huh7 Vector group. These results of xenograft experiments confirmed that Cav-1 expression was enhanced by GLI1 and contributed to GLI1-driven EMT phenotype in HCC.

## Discussion

HCC is the leading cause of cancer-death worldwide[27]. Surgical treatment including hepatic resection and liver transplantation at the early stage is the only curative therapy for HCC patients. Unfortunately, due to high frequency of intrahepatic and extrahepatic metastasis, most of HCC patients are diagnosed at the advanced stage and not suitable to receive the curative therapy mentioned above, which leads to poor prognosis of HCCs. Hence, it is urgent to explore the molecular mechanism of HCC progression and discover the novel HCC markers and target agents.

Cav-1 is one of primary structural components of caveolae and located in both cell membrane and cytoplasm. Cav-1 is reportedly responsible to multiple cell signal transduction which is critical to the determination of cell fate. A growing body of evidences have suggested that Cav-1 is overexpressed and associated with metastatic progression of various cancers including prostate cancer[28], lung cancer[29], renal cancer[30] and esophageal cancer[8]. There is a controversial about Cav-1 expression on HCC and its role on the prognosis of HCC. In this study, after collecting specimens from HCCs and the relevant clinical information and analyzed them, we found that Cav-1 was up-regulated in HCC tissues significantly and predicted worse survival after hepatic resection, which is consistent with the common results of most groups. These data support that Cav-1 could function as an oncogenic protein in HCC. To date, the mechanism of overexpression of Cav-1 in cancer remains unclear. Cokakli and his colleagues[18] found that Cav-1 could induce EMT phenotype of HCC cells. And our previous study showed that GLI1 affected EMT phenotype of HCC through modulating multiple cell signaling pathways[24]. Thus, we wondered whether Cav-1 was up-regulated by GLI1 and consequently induced EMT of HCC cells. Here, our results showed that enforced expression of GLI1 increased Cav-1 expression and silencing GLI1 repressed Cav-1 expression in HCC cells in turn, which initially proves the regulatory effect of GLI1 on Cav-1 expression in HCC cells. Then, we attempted to find out the relationship between GLI1 and Cav-1 expression on HCC tissues from HCC patients undergoing hepatic resection by IHC and found that Cav-1 expression was significantly associated with GLI1 expression positively, which confirms the regulatory function of GLI1 on Cav-1 in HCC. In additional, we established HCC xenograft mouse model and found that Cav-1 was expressed positively in xenografts with high GLI1 level and disappeared in xenografts without GLI1 expression. These results identify conclusively that Cav-1 is up-regulated by GLI1 in HCC. However, Cav-1 was found not to regulate the markers of Hh signaling activation (PTCH1 and SNAI1) by Western immunoblotting and affect Hh signaling activation by the Hh signal reporter assay in this investigation, which prompts that Cav-1 doesn't result in Hh signaling activation and up-regulation of GLI1 consequently. We did not find the consensus GLI1 binding sites (GACCACCCA) on the promoter of Cav-1 sequence. Although there were two potential GLI1 binding sites found in the promoter of Cav-1 with low homology to the canonical GLI1 binding sequence and Cav-1 luciferase reporter assay in this study also showed that forced expression of GLI1 leaded to up-regulation of Cav-1 promoter activity, we failed to prove the direct regulatory function of GLI1 on Cav-1 expression in HCC cells by ChIP assay. The mechanism of regulatory effect of GLI1 on Cav-1 expression in HCC remains indistinct.

In the present investigation, we enhanced Cav-1 expression in Huh7 cells and obtained typical EMT phenotype including higher cell mobility, higher invasion capacity, less epithelial marker expression and more expression of mesenchymal markers, which is consistently with the results of Cokakli group[18]. On the other hand, we silenced Cav-1 expression in two kinds of HCC cells (SNU449 cells and SK Hep1 cells) and reversed the EMT phenotype of both HCC cells. These data suggest strongly that Cav-1-induced EMT exists widely in HCC. To establish the role of Cav-1 on GLI1-induced EMT of HCC cells, we suppressed Cav-1 up-regulation induced by GLI1 and consequently abolished EMT induced by GLI1 in Huh7 cells. In the *in vivo* experiements, both obvious EMT phenotype and Cav-1 up-regulation were detected in xenografts from Huh7 GLI1 group, which indicates that Cav-1 is involved in HCC EMT induction by GLI1 *in vivo*. Interestingly, SNAI1 established by several groups including us to take part in EMT induction by GLI1 was found not to be up-regulated by Cav-1 *in vitro* in this investigation, whereas Twist, another well-known EMT inducer of HCC, was found up-regulated by Cav-1, which points out that GLI1 could induce HCC cell EMT through both the GLI1-SNAI1 axis and the GLI1-Cav1-Twist cascade. These findings make clear that Cav-1 plays an important role in GLI1-driven EMT phenotype in HCC cells. And the data of in *vivo* experiments also showed that GLI1 promoted tumor xenograft growth significantly, which verifies the oncogenic effect of GLI1 on HCC further.

Taken together, there are four main findings in the present study: 1. Cav-1 is up-regulated in HCC and predicts poor prognosis after radical hepatic resection; 2. GLI1 is attributed to up-regulation of Cav-1 in HCC; 3. Cav-1 plays a critical role on GLI1-induced EMT phenotype of HCC cells; 4. Besides the GLI1-SNAI1 axis, GLI1 is able to induce EMT phenotype of HCC cells via the GLI1-Cav1-Twist cascade. These findings suggest that Cav-1 is a potential mark of HCC progression and promising therapy target.

## Materials and Methods

### Ethics Statement

Written informed constent was obtained from all patients recruited in this study. The ethics committee of our hospital approved all protocols according to the Helsinki Declaration of 1975. And all experimental protocols were approved by the institutional animal care and use committee of our hospital.

### Materials

DMEM medium, RPMI 1640 medium, FBS and trypsin/EDTA were purchased from Invitrogen Co. (Carlsbad, CA, USA). The GLI1 expressing plasmid was constructed by cloning GLI1 cDNA into the pCMV-Tag2B vector from Stratagene (Santa Clara, CA). Cav-1 cDNA was cloned into the pCMV-Tag2B vector to construct Cav-1 expressing plasmid. Both GLI1 siRNAs (Catalog No.: sc-37911) and Cav-1 siRNAs (Catalog No.: sc-29241) were from Santa Cruz Biotechnology, Santa Cruz, CA, USA). The 18s rRNA TaqMan probe (Hs99999901_s1), Cav-1 TaqMan probe (Hs00971716_m1) and GLI1 TaqMan probe (Hs01110766_m1) were purchased from Applied Biosystems (Carlsbad, CA, USA). The rabbit polyclonal GLI1 antibody, rabbit polyclonal Cav-1 antibody, rabbit polyclonal SNAI1 antibody, rabbit polyclonal Slug antibody and rabbit polyclonal PTCH1 antibody were from Cell signaling (Danvers, MA, USA). The mouse monoclonal E-cadherin antibody and rabbit polyclonal N-cadherin antibody were both purchased from Santa Cruz Biotechnology. The rabbit polyclonal Twist antibody was from Abcam (MA, USA). The rabbit polyclonal Fibronectin antibody, mouse monoclonal Vimentin antibody, mouse monoclonal β-actin antibody and 4,6-diamidino-2-phenylindole (DAPI) were from Boster Biotechnology (Wuhan, China).

### Cell Culture

4 HCC cell lines (Hep3B, HepG2, SKHep1 and SNU449) were obtained from the American Type Culture Collection (Manassas, VA, USA) and Huh7 cell line was a kind gift from Prof. Kefeng Dou (Department of Hepatobiliary Surgery, Xijing Hospital, Fourth Military Medical University). Both SK Hep1 cells and Hep3B cells were both cultured in complete MEM medium with 10%FBS. Huh7 cells were grown in DMEM medium with 10% FBS. SNU449 and HepG2 cells were cultured in RPMI 1640 medium with 10% FBS.

### Establishment of Stable Transfectant Clones

GLI1 expressing plasmid or Cav-1 expressing plasmid was transfected into Huh7 cells using FuGENE6 transfection reagent from Promega (Madison, WI, USA) as GLI1-expressing Huh7 cells (Huh7 GLI1 cells) or Cav-1-expressing Huh7 cells (Huh7 Cav-1 cells). The pCMV-Tag2B vector was also transfected into Huh7 cells as Huh7 vector control cells (Huh7 Vector cells). Stable transfection clones were obtained after 2-week selection with Geneticin (G418) from Invitrogen (Carlsbad, CA, USA) at a dose of 500 µg/mL.

### RNAi Transfections

SiRNAs against GLI1 or Cav-1 and a scrambled siRNAs were both obtained from Santa Cruz Biotechnology (Santa Cruz, CA, USA). Another pool of different siRNA constructs against Cav-1 and the relevant scrambled siRNAs were purchased from GenePharma Co. (Shanghai, China) to confirm further the role of Cav-1 in HCC progression. HCC cells were seeded in six-well plates at the concentration of 0.2×10^6^ per well and cultured overnight. Then the cells in each well were transfected with 100 nM siRNAs using Lipofectamine RNAi MAX Reagent (Invitrogen, CA, USA) according to the manufacture's instructions. At 48 h after transfection, the cells were ready for further assessment.

### Quantitative Real-time Reverse Transcription Polymerase Chain Reaction (qRT-PCR)

Total RNA was extracted from HCC cell lines with the Rneasy kit from Qiagen Co. (Valencia, CA, USA). Reverse transcription was carried out to synthesize cDNA using the High Capacity cDNA Reverse Transcription Kit from Applied Biosystems (Carlsbad, CA, USA). QRT-PCR was performed using ABI TaqMan Gene Expression assays in an ABI 7300 system using TaqMan probes targeting different genes. GLI1 expressing plasmid was used to make the standard curve as standard sample and 18s rRNA was regarded as the internal control. The mRNA levels of both GLI1 and Cav-1 were normalized to 18s rRNA mRNA level in the same sample. All assessments were repeated three times dependently.

### Western Immunoblotting

The protocol of Western immunoblotting has been described in our previous paper[26]. Briefly, 30 µg denatured protein samples were divided by polyacrylamide gel electrophoresis (PAGE). Then protein blots were transferred into PVDF membrane and incubated overnight with the primary antibodies respectively at 4°C. After washed 3 times by tris-buffered saline buffer with tween (TBST), blots were then detected with the relevant secondary antibodies conjugated with HRP, and signals were visualized using the HyGLO HRP detection kit from Denville (Metuchen, NJ, USA). *β*-actin was measured to control for equal loading.

### Wound Healing Assay

HCC cells were seeded into 6-well plates and cultured to more than 90% confluency. Scratch wound were made using a 1000-µL-pipette tip. Pictures of wounds were taken with a phase-contrast microscope at 0, 24 and 48hours. Cell migration was quantitated by assessing the width of the wounds. Each experiment was repeated three times.

### Invasion Assay

Millicell invasion chambers (8 µm pore size) from Millipore Corp were used for invasion assay. Huh7 cells was cultured in serum-free medium for 24hours and then seeded into the upper chamber precoated with basement membrane Matrigel. The upper chamber was filled with serum-free medium and meanwhile medium with 20% FBS was placed into bottom chamber. These cells were grown at the normal condition for 24hours. Next, the cells remaining in the top surface of Matrigel membrane was swabbed carefully and the membrane was fixed with paraformaldehyde for 10 min. The cells in the membrane was stained by crystal violet solution and detected under microscope. Five fields were chosen randomly to get the mean cell number in each membrane. Each exanimation was repeated six times.

### *In Vivo* Experiments

Twelve 4- to 6-week old male nude mice were divided into two groups randomly: Huh7 GLI1 group and Huh7 Vector group. Two million Huh7 GLI1 cells or Huh7 Vector cells were suspended in 150 µL of Matrigel. The cell mixture was injected subcutaneously into the flanks of nude mice. We measured tumor sizes with calipers every three days. Mice were sacrificed at the 15th day after cell injection. IHC staining was performed in xenograft tissues to detect the expression of both GLI1 and Cav-1. All experimental protocols were approved by the institutional animal care and use committee of our hospital.

### Immunohistochemistry Staining

HCC tissues and adjacent tissues (>2 cm distance to the resection margin) were obtained from 80 HCCs undergoing liver resection in our hospital from 2009 to 2010 and kept in paraformaldehyde. All patients did not receive chemotherapy or embolization therapy before operation. Clinical data were collected from the medical records. Written informed constent was obtained from all patients recruited in this study. The ethics committee of our hospital approved all protocols according to the Helsinki Declaration of 1975.

IHC staining was performed as described previously[25]. Briefly speaking, the paraffin slides were dewaxed and dehydrated. Endogenous peroxidase activity was blocked for 30 min with a methanol solution containing 0.3% hydrogen peroxide. Citrate buffer was used to retrieve antigen and then slides were incubated with the primary antibody overnight at 4°C. After washing slides with PBS three times, we incubated slides with the secondary antibody. The slides were stained by the avidin-biotin-peroxidase complex (SABC) method. The slides were visualized with diaminobenzidine and counterstained with hematoxylin. Finally, tissues were dehydrated in alcohol and xylene.

All slides were observed by two pathologists independently to assess the Edmonson classification and the staining results of IHC. The results of IHC staining were expressed by an immunohistochemical score and the percentage of tumor cells showing specific immunoreactivity. Staining intensity was classified into four grades: 0, none; 1, weak; 2, moderate; 3, strong. The percentage of positive cancer cells was also divided into four grades: 0, <5%; 1, 6–25%; 2, 26–50%; 3, 51–75%; 4, >75%. The total score of >1 were defined as positive staining. Each slides were observed for 10 independent high magnification field (×400) to get the mean staining score.

### Hh Signaling Reporter Assay

GLI-Luciferase reporter plasmid containing eight consecutive GLI-binding sites downstream of the luciferase gene was used for Hh signaling reporter assay. The GLI-Luciferase reporter plasmid is a gift from Prof. Lewis Roberts (Devision of Gastroenterology, Mayo Clinic, MN). Briefly, HCC cells plated in six-well plates at 60% confluency were transfected with GLI reporter plasmid with the help of the FuGENE6 transfection reagent. Luciferase activity was assessed using the Dual-Luciferase Reporter assay from Promega (Madison, WI) and normalized by protein quantification. Each experiment was repeated six times.

### Statistical Analysis

All data were expressed as means and standard errors of the mean. Differences between groups were compared with the Mann-Whitney test or Student-t test. The relationship between the clinical features of HCC patients and Cav-1 expression in HCC tissues was analyzed by Spearman rank test. The Spearman rank test was also used to analyze the association between GLI1 expression and Cav-1 expression in HCC tissues of both patients and HCC xenograft. Differences of the Kaplan-Meier curves between Cav-1 high group and Cav-1 low group was analyzed using the log rank test. A P value of <0.05 was used for significance. All statistical analysis was performed using PRISM 4 (Graphypad, La Jolla, CA, USA).

## Supporting Information

Figure S1 **Cav-1 didn't mediate activation of Hh signaling in HCC cells.** (A) Overexpression of Cav-1 in Huh7 cells didn't result in up-regulation of PTCH1, Slug and SNAI1, while knockdown of Cav-1 didn't affect the expression of PTCH1, Slug and SNAI1 in both SNU449 cells and SK Hep1 cells; (B) Overexpression of Cav-1 in Huh7 cells didn't lead to more activation of Hh signaling; (C) Knockdown of Cav-1 didn't affect activation of Hh signaling in both SNU449 cells.
(TIF)

Figure S2 **Knockdown of Cav-1 by another pool of Cav-1 siRNAs decreased migration and invasion capacities of HCC cells.** (A) Another siRNA sequences against Cav-1 from GenePharma Co. (Cav-1 siRNA 2) was verified to decrease Cav-1 expression successfully in SNU449 cells by qRT-PCR and western immunoblotting; (B) Cav-1 siRNA 2 transfection resulted in remarkable down-regulation of migration capacity of SNU449 cells; (C) Invasion ability of SNU449 cells was repressed apparently after Cav-1 siRNA 2 transfection.
(TIF)

Figure S3 **GLI1 overexpression promoted transcriptional activity of Cav-1 promoter.** (A) Location and sequences of two potential GLI1-binding sites in the promoter of Cav-1; (B) Huh7 cells were transfected with Cav-1 promoter reporter plasmid, together with either control vector (Huh7 Vector) or GLI1 expressing vector (Huh7 GLI1), and it was shown that there was more reporter activity in Huh7 GLI1 cells than one in Huh7 Vector cells.
(TIF)
