# Supplementary figures and images for: Caveolin-1 Is Up-Regulated by GLI1 and Contributes to GLI1-Driven EMT in Hepatocellular Carcinoma
Source: PLoS One. 2014 Jan 13;9(1):e84551. doi: 10.1371/journal.pone.0084551 (PMC3890273; doi:10.1371/journal.pone.0084551)

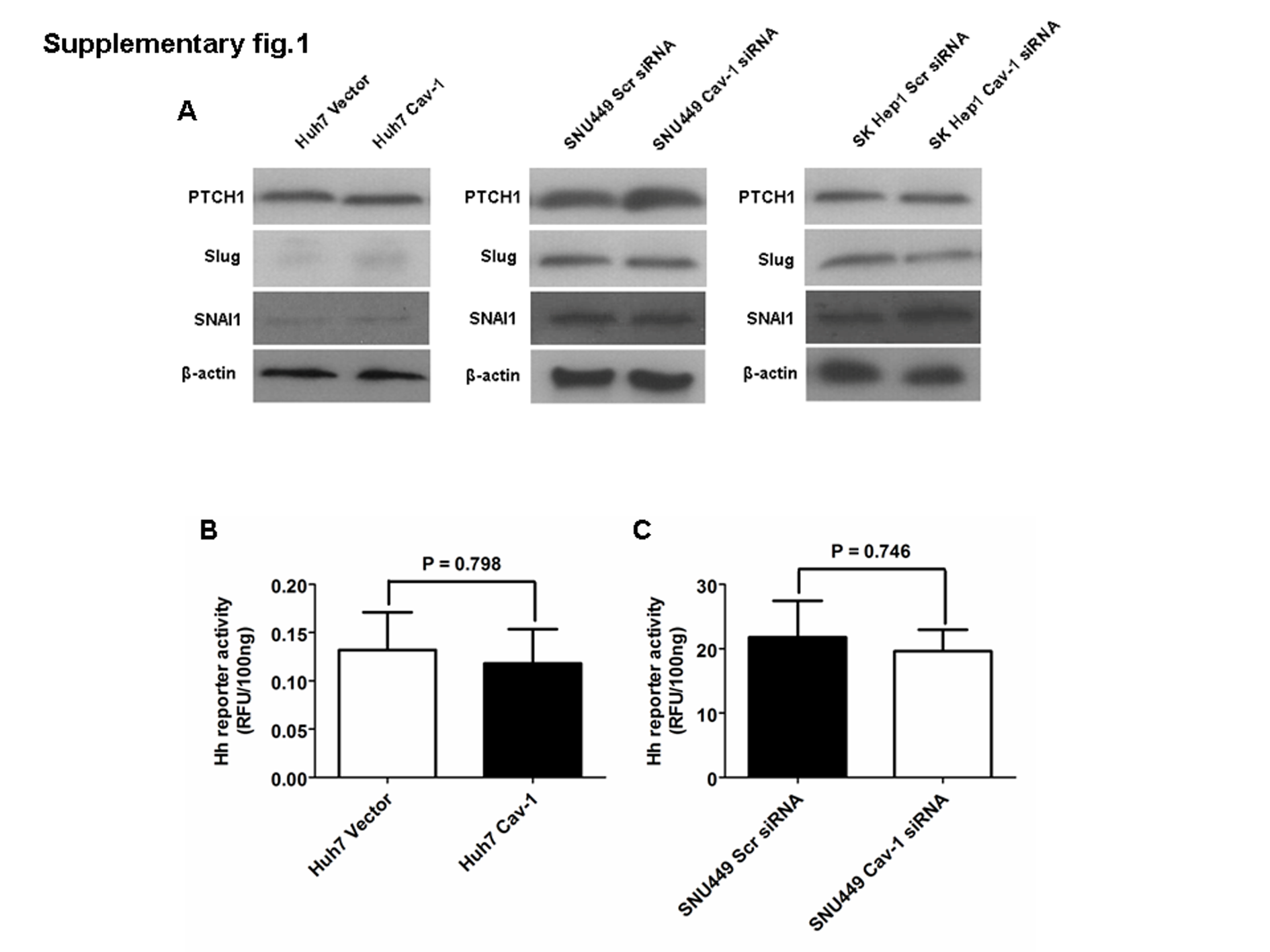

Supplement: Figure S1 — Cav-1 didn't mediate activation of Hh signaling in HCC cells. (A) Overexpression of Cav-1 in Huh7 cells didn't result in up-regulation of PTCH1, Slug and SNAI1, while knockdown of Cav-1 didn't affect the expression of PTCH1, Slug and SNAI1 in both SNU449 cells and SK Hep1 cells; (B) Overexpression of Cav-1 in Huh7 cells didn't lead to more activation of Hh signaling; (C) Knockdown of Cav-1 didn't affect activation of Hh signaling in both SNU449 cells. (TIF) [file pone.0084551.s001.tif]

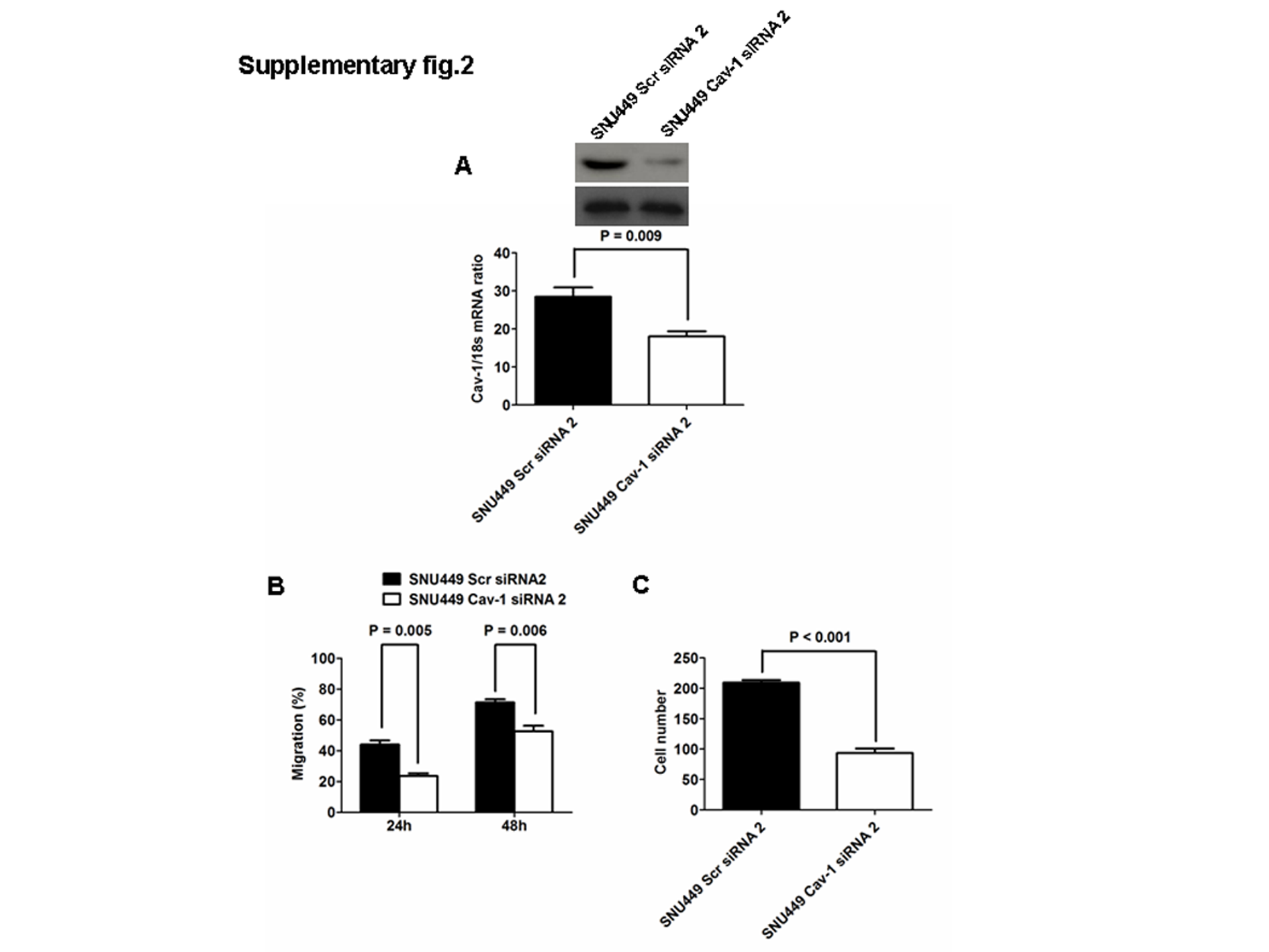

Supplement: Figure S2 — Knockdown of Cav-1 by another pool of Cav-1 siRNAs decreased migration and invasion capacities of HCC cells. (A) Another siRNA sequences against Cav-1 from GenePharma Co. (Cav-1 siRNA 2) was verified to decrease Cav-1 expression successfully in SNU449 cells by qRT-PCR and western immunoblotting; (B) Cav-1 siRNA 2 transfection resulted in remarkable down-regulation of migration capacity of SNU449 cells; (C) Invasion ability of SNU449 cells was repressed apparently after Cav-1 siRNA 2 transfection. (TIF) [file pone.0084551.s002.tif]

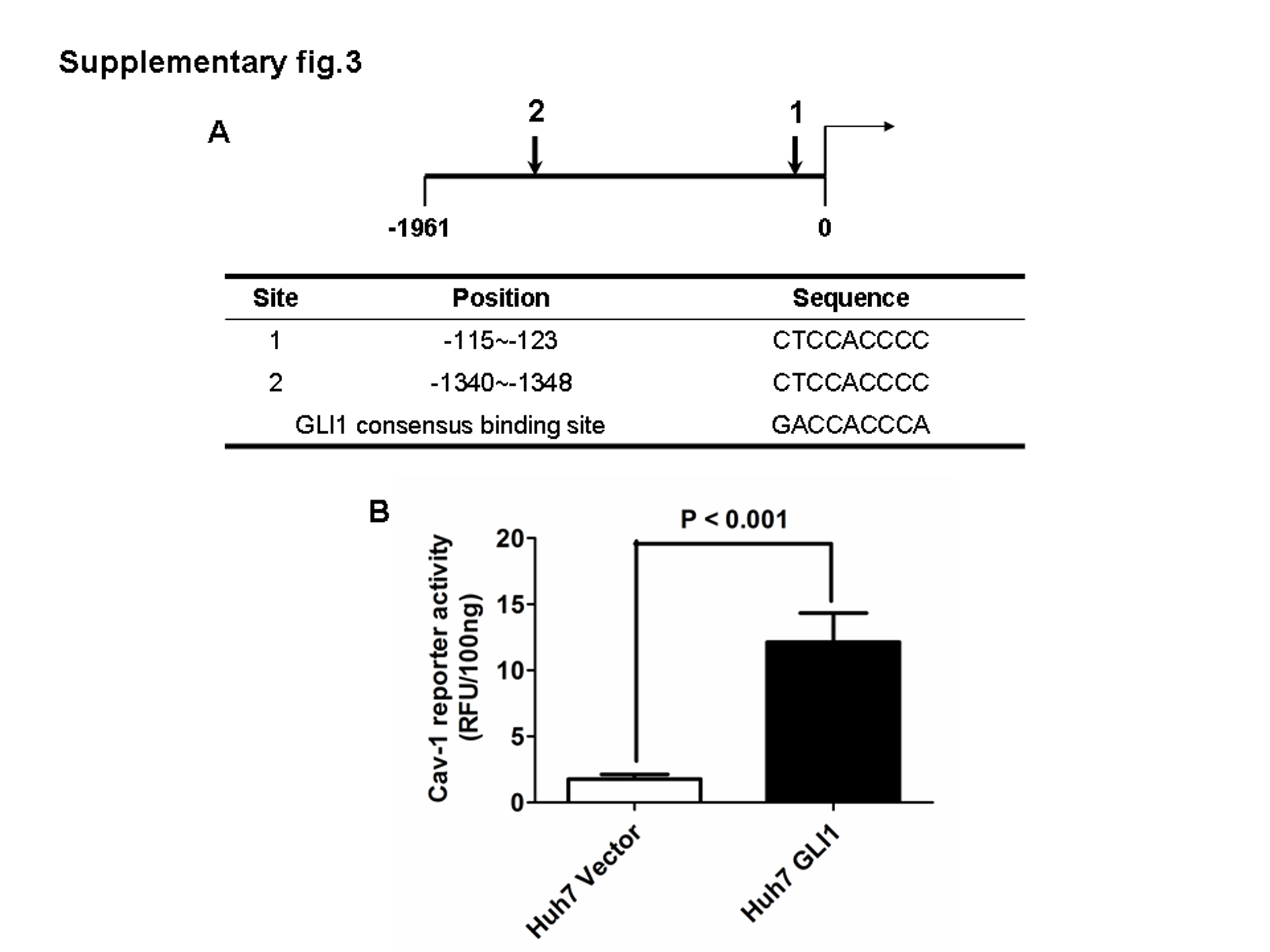

Supplement: Figure S3 — GLI1 overexpression promoted transcriptional activity of Cav-1 promoter. (A) Location and sequences of two potential GLI1-binding sites in the promoter of Cav-1; (B) Huh7 cells were transfected with Cav-1 promoter reporter plasmid, together with either control vector (Huh7 Vector) or GLI1 expressing vector (Huh7 GLI1), and it was shown that there was more reporter activity in Huh7 GLI1 cells than one in Huh7 Vector cells. (TIF) [file pone.0084551.s003.tif]
